# Influence of Different Bacteria Inocula and Temperature Levels on the Chemical Composition and Antioxidant Activity of Prickly Pear Vinegar Produced by Surface Culture

**DOI:** 10.3390/foods11030303

**Published:** 2022-01-24

**Authors:** Ikram Es-sbata, Remedios Castro, Yolanda Carmona-Jiménez, Rachid Zouhair, Enrique Durán-Guerrero

**Affiliations:** 1 Analytical Chemistry Department, Faculty of Sciences-IVAGRO, University of Cadiz, Agrifood Campus of International Excellence (CeiA3), Polígono Río San Pedro, s/n, 11510 Puerto Real, Cadiz, Spain; ikram.essbataess@alum.uca.es (I.E.-s.); remedios.castro@uca.es (R.C.); yolanda.carmona@uca.es (Y.C.-J.); 2 Laboratory of Plant Biotechnology and Molecular Biology, Department of Biology, Faculty of Sciences, Moulay Ismail University, Meknes 11201, Morocco; r.zouhair@umi.ac.ma

**Keywords:** prickly pear, vinegar, fermentation, thermotolerant bacteria, volatile compounds, polyphenolic compounds

## Abstract

This work intends to determine the effect on the aroma profile, phenolic content and antioxidant activity of prickly pear vinegars produced by the surface culture at two different fermentation temperatures and using different acetic acid bacteria (AAB) inocula. Prickly pear wine was fermented at two temperature levels (30 and 37 °C) by using bacteria inocula containing *Acetobacter*, *Gluconobacter* or a mixture of bacteria isolated from Sherry vinegars. Eighty-five individual volatile compounds from different families and sixteen polyphenolic compounds have been identified. It was confirmed that the highest temperature tested (37 °C) resulted in a lower concentration of volatile compounds, while no significant effect on the vinegars’ volatile composition could be associated with the AAB inoculum used. Contrariwise, the highest content of polyphenolic compounds was detected in those vinegars produced at 37 °C and their concentration was also affected by the type of AAB inoculum used. Prickly pear wine displayed greater antioxidant activity than juices or vinegars, while the vinegars obtained through the mixture of AAB from Sherry vinegar showed higher antiradical activity than those obtained through either of the two AAB genera used in this study. It can be therefore concluded that, although the volatile content of vinegars decreased when fermented at a higher temperature, vinegars with a higher content in polyphenols could be obtained by means of partial fermentations at 37 °C, as long as thermotolerant bacteria were employed.

## 1. Introduction

*Opuntia ficus-indica (L). Mill*. is a plant that belongs to the Cactaceae family and can grow in arid and semi-arid climates. Its common name is prickly pear, nopal cactus or cactus pear. It originally comes from tropical or subtropical American regions, but it has already been naturalized on all continents [1]. In Morocco, around one hundred and fifty thousand hectares of area are cultivated with *O. ficus-indica* and the annual production of prickly pear under optimal conditions can reach up to 20 tons dry matter/hectare/per year [2]. The prickly pear fruit pulp is considered to be the most edible and interesting part to be processed for alimentary purposes. These fruits contain health-promoting substances, since they are a good source of nutrients such as polyphenols and betalains [3]. The biochemical composition of cactus fruits changes over their development and ripening. This depends not only on the plant variety but also on the color, number of seeds, size, sugars, fats, proteins, pectins and non-volatile organic acids contents [4]. 

Vinegar is a food condiment that is worldwide produced from a variety of raw materials, that can be vegetables, fruits or grains. Many types of vinegar are currently commercialized, including balsamic, rice, wine or black vinegar. These vinegar varieties present unique characteristics, flavors and tastes depending on the raw material used for their production, the fermenting procedures and the microorganisms involved in the process [5]. 

Prickly pear juice, because of its high sugar content and low acid blend, seems to be an interesting alternative for the production of vinegar [2]. Obtaining vinegar from cactus pear juice would be a new way to valorize cactus fruit through a simple process that can be performed at different industrial scales [6]. Two vinegar production methods are generally recognized, the slow traditional or surface method and the quick industrial or submerged one [7]. Both the method employed, as well as the fermentation time allowed, have an impact on vinegar quality. Vinegars obtained through the traditional method usually have a better sensory quality, with particularly richer aromas than those of the vinegars elaborated using the industrial method. On the other hand, the submerged method is a more economical process that leads to greater yields and is therefore generally preferred by industrial producers [8,9]. Vinegar quality is the end result of a number of factors, such as raw material, acetification conditions and acetic acid bacteria metabolism, which involves the production of acetic acid from ethanol as well as other transformation processes such as oxidations or the formations of esters, among others [10].

A number of studies have already revealed how important the production method is with respect to the vinegar’s final aroma profile and hence to their organoleptic qualities [9,11]. Beside the production method, there are other parameters that exert an influence on the proportion of certain compounds, such as volatile and phenolic compounds, which are key to determining vinegar quality—certain acetification parameters, such as the amount of oxygen, the optimum temperature and the substrate loading, are some of these key indicators [12]. In order to control and optimize vinegar quality, some recent studies on fruit vinegar production have focused on the isolation of certain acetic acid bacteria, on the processing conditions and on the vinegars’ phenolic and aromatic profiles [13]. Apart from the above-mentioned parameters, vinegar production using different bacteria strains, depending on their tolerance to acetic acid high concentrations, has been tested [14]; thus, at lower concentrations of acetic acid in the medium, certain species of the *Acetobacter* genus predominate, where *Acetobacter aceti* is generally the most often employed for vinegar production [15]; however, when the acetic acid content reaches over 5%, the species from the *Gluconacetobacter* genus are more effective [14,16,17]. Moreover, both high temperature and high ethanol concentrations have an evident effect on vinegar fermentation processes; thus, thermotolerant and ethanol-resistant strains are expected to become feasible technologies for effective vinegar fermentation under unfavorable acetification conditions.

Each vinegar's sensory characteristics, as well as its different flavors and aromas, are determined by the pungent flavor of the acetic acid used for its production. Organic acids, essential amino acids, vitamins, minerals, volatile compounds and other fermentation products also play a major role regarding the organoleptic properties of vinegars [18]. 

Vinegar aroma, in particular, which depends on a large number of volatile compounds, is one of its most determinant characteristics with regard to food quality and consumer acceptance. These compounds may either be present in the raw material itself or may be generated over the production process [19]. In the particular case of prickly pear fruit vinegar, alcohols and esters are considered to be key aroma substances, responsible for its characteristic faint melon or cucumber-like aroma. 2-(E/Z)-2,6-nonadien-1-ol and 2-methylbutanoic acid methyl ester have been identified as the volatile compounds that confer cactus fruit vinegar with their distinctive aroma [20]; however, in a recent study, Farag et al. reported that the predominant volatile compounds in this fruit were short-chain aldehydes (25–32%) and acids (25–29%) [21].

Phenolic compounds, on the other hand, seem to be secondary metabolites that are closely related to the color and flavor traits of fruits, juices, and wines [22]. Some recent studies on *Opuntia* spp. stated that cactus pear fruit is a good candidate to develop new healthy food, because of their high content of biofunctional polyphenolic compounds [23,24]; thus, the polyphenolic fingerprint of prickly pear products is mainly characterized by the presence of flavonols and phenolic acids [25,26]. Particularly, among the minor compounds in *O. ficus-indica*, betalains and polyphenols seem to be the most valuable antioxidants with regard to the nutritional quality of prickly pears and of their transformation products [27]. Other studies have reported that prickly pear juice is rich in phenolic compounds that act as effective radical scavengers [28]. Several polyphenolic compounds, mainly represented by ferulic acid derivative, rutin, gallic acid and catechin, have been identified in prickly pear pulp [29,30]. A simple way to evaluate the global antioxidant potential of a food, due to the presence of the different bioactive substances present such as polyphenols, is the determination of its antioxidant activity. Although there are clear differences between the antioxidant activity in vitro and in vivo [31], the in vitro measurement could give an initial idea of the possible healthy character of a certain food product.

In view of all the above said, this study intends to investigate and evaluate, on the one hand, the effect of different fermentation temperatures and bacteria inocula on the concentration of volatile and phenolic compounds in final prickly pear vinegars, and, on the other, to determine the antioxidant activity of a number of prickly pear samples. In the present study, three types of acetic acid bacteria inocula have been used to accomplish the fermentation of a Moroccan prickly pear wine to produce prickly pear vinegar under two temperatures (30 and 37 °C), all of them according to a surface culture production method. To the best of our knowledge, this is the first work that deals with the analytical characterization of prickly pear vinegars produced under different surface culture conditions. 

## 2. Materials and Methods

### 2.1. Fruit

The prickly pear fruits (yellow-orange color) from wild cultivars grown in Marrakesh-Safi-Morocco region (coordinates: 31°37′ N 8°0′ W) were harvested during August (2019). Each fruit piece was manually peeled off and the pulp was weighed and preserved under frozen storage (−80 °C) until further procedures. The prickly pear fruit pulps were mechanically crushed using a conventional household blender. Then, the pulp mixture was homogenized and filtrated using a colander (0.5 mm mesh size) in order to separate the juice from the seeds. The juice was stored at 4 °C until its analysis. The °Brix, °Baumé, pH and density values of the final juice were measured at 20 °C by means of a DMA 4500 M densimeter (Anton Paar, Graz, Austria).

### 2.2. Alcoholic Fermentation

The alcoholic fermentation was performed at a pilot scale using stainless-steel tanks covered by mosquito nets to avoid any contamination by insects. Twenty-five liters of the final prickly pear juice were fermented in duplicate (50 L total volume) and the fermentation temperature was maintained at a constant 22 °C to avoid any losses of volatile compounds during the process. 60 mg/L of total sulfur dioxide (potassium metabisulphite, Agrovin, Spain) was added to prevent the growth of undesirable microorganisms. A total of 0.35 g/L of diammonium phosphate was also added as a nutrient to the matrix; 0.20 g/L of *Saccharomyces cerevisiae* active dry yeast, previously activated at 35 °C for 20 min were also inoculated to start the fermentation process (Enartis Ferm SB, Trecate, Italy). The process was monitored by measuring the sugar content in the substrate. After the fermentation had been started, the sugar content was increased until 14 °Brix degrees was reached (equal to the initial °Brix value of the juice) by adding commercial white refined beet sugar (AB Azucarera Iberia, Madrid, Spain) suitable for human intake, to rise the final alcoholic degree. Then, the prickly pear wine produced was centrifuged at 15,000× *g* for 10 min and stored at 4 °C.

### 2.3. Acetic Fermentation

#### 2.3.1. Bacterial Preparation

Ten thermo-ethanol tolerant strains (5 *Acetobacter* and 5 *Gluconobacter;* AAB) were previously isolated from Moroccan prickly pear fruit following the procedure described in the previous work [32]. Further, a mixture of strains from an unfiltered Sherry vinegar (Jerez, Spain) was used in order to compare the different acetification profiles obtained from different bacteria inocula. The selected AAB were precultured in a broth medium and incubated at 30 °C for 24 h with continuous and vigorous agitation in order to initiate the acetic acid bacterial cells' rapid proliferation. When the measured optical density (OD 600 nm) of the suspension reached over 1.2, the cells with 10% (*v*/*v*) of inoculum were transferred into the prickly pear wine to start the acetic fermentation according to the traditional method.

#### 2.3.2. Surface Culture Acetification

The acetification of the prickly pear wine through surface culture was carried out in 500 mL Erlenmeyer flasks which had been previously sterilized and covered with cotton sheets. The flasks were filled with 250 mL of prickly pear wine and each one of them was then inoculated with 10% (*v*/*v*) of the different types of AAB inocula. The experiments were conducted at 30 °C and 37 °C in duplicate and the flasks were not agitated to allow the atmospheric oxygen to diffuse slowly into the fermenting medium. A temperature of 37 °C showed the best results in terms of production of acetic acid by thermotolerant bacteria in a previous work [32] and it was compared with the usual acetification temperature (30 °C). The content in each flask was sampled every three weeks and each sample’s total acidity was determined by titration with NaOH using phenolphthalein indicator. The total acidity content was expressed as g of acetic acid/100 mL of vinegar. When the acidity content stopped increasing, the fermentation process was considered as completed.

### 2.4. Analysis of Volatile Compounds

#### 2.4.1. Sample Preparation

The samples were analyzed by SBSE/GC-MS according to the method described by Durán Guerrero et al. [19]. The volatile compounds from the vinegar samples, were extracted by means of commercially available polydimethylsiloxane stirs bars, 10 mm length x 0.5 mm film thickness (supplied by Gerstel, Mülheim a/d Ruhr, Germany). For each stir bar sorptive extraction (SBSE) analysis, a volume of 25 mL of the sample was pipetted and placed into a 100-mL Erlenmeyer flask and 5.85 g of NaCl (Scharlau, Barcelona, Spain) was added and dissolved by agitation. Then, 84 µL of 4-methyl-2-pentanol solution (Sigma, Steinheim, Germany) (2.27 g/L in Milli-Q water containing 80 g/L of acetic acid) were also added to the sample. The Erlenmeyer flasks were placed on a magnetic stirrer (Gerstel), and they were stirred at 1250 rpm at 25 °C for 120 min. Then, the stir bars were removed from the vinegar samples and placed for a few seconds in distilled water, in order to remove the NaCl as well as any remains of prickly pear pulp that might have stuck onto them. They were then gently dried using a lint-free tissue. Finally, the dried stir bars were transferred into a glass thermal desorption tube and they were thermally desorpted.

#### 2.4.2. Instrumentation

The thermal desorption system used for the coated stir bars was a commercial TDS-2 thermal desorption unit (Gerstel) connected to a programmed-temperature vaporization (PTV) injector CIS-4 (Gerstel) through a heated transfer line. The PTV was installed onto the chromatographic system. The thermo-desorption unit was equipped with an MPS 2L autosampler (Gerstel) with the capacity to handle 98 coated stir bars. The desorption temperature was configured to go from 40 °C up to 300 °C (held for 10 min) at 60 °C/min under a 75 mL/min helium flow and the desorbed analytes were cryofocused using the PTV system with liquid nitrogen at −140 °C. Finally, the PTV system was configured to go from −140 °C up to 300 °C (held for 5 min) at 10 °C/s and the analytes were analyzed by Gas Chromatography–Mass Spectrometry (GC-MS). An Agilent 6890 GC-5973N MS system (Agilent, Little Falls, DE, USA), equipped with a DB-Wax capillary column (J&W Scientific, Folsom, CA, USA), 60 m × 0.25 mm i.d. with a 0.25 µm coating, which was used on the electron impact mode to perform the capillary GC–MS analysis. A 1.0 mL/min helium flow was used as the carrier gas. The peaks were identified by means of the Wiley library according to their mass spectra analogies (>85% matching) and confirmed by the retention times of the standards, when available, or by the retention data found in the literature. The linear retention index of each one of the compounds was determined by means of a DB-Wax polar column and compared against those reported in the literature [33,34,35]. The semi-quantitative data were obtained by measuring the relative base of the ion peak area in relation to that of 4-methyl-2-pentanol, as the internal standard. All the analyses were performed in duplicate.

### 2.5. Analysis of Phenolic Compounds

The phenolic compounds in the prickly pear samples were identified and quantified by means of a Waters Acquity UPLC system (Waters Corps. Milford, MA, USA), equipped with a diode array detector (DAD) and following the method proposed by Schwarz et al. [36]. An Acquity UPLC BEH C18 column (100 × 2.1 mm/ID, with 1.7 μm particle size), also from Waters, was used. All the samples (juice, wine and vinegars) were previously filtered through 0.22 μm nylon filters manufactured by Scharlab (Barcelona, Spain).

The phenolic compounds were identified by comparing retention times and ultraviolet-visible (UV-VIS) spectra against those of their corresponding commercial standards (Fluka, Buchs, Switzerland; Sigma, Steinheim, Germany; and East Kodak, Rochester, NY, USA). Each identified compound was quantified by comparison against the calibration curve obtained from their corresponding standard at 280 nm (for gallic acid, hydroxy-tyrosol, epigallocatechin, catechin, tyrosol, vanillic acid, syringic acid, ethyl gallate, *m*-coumaric acid, hesperidin and naringenin), 320 nm (for protocatechualdehyde, *p*-coumaric acid, ferulic acid, quercetin and cinnamic acid) and 255 nm (for *p*-hydroxybenzoic acid) at seven concentration levels, except for hydroxy-tyrosol, which was quantified as tyrosol. All the analyses were conducted in duplicate.

### 2.6. Analysis of the Antioxidant Activity

The antioxidant activity levels of the prickly pear juice, the wine and the different vinegars were determined by DPPH (1,1-diphenyl-2-picrylhydrazyl) according to the method reported by Carmona-Jiménez et al. [37]. A total of 200 µL of sample or ethanol (blank) were added into vials containing 3.3 mL of a 50 µM solution of DPPH in ethanol prepared daily (0.069 ppm of the initial DPPH). Then, the mixture was allowed to sit at room temperature for 3 h, the absorbance at 515 nm was measured using a Cary 50 Bio spectrophotometer (Varian, Australia). All the measurements were conducted in duplicate. The exact concentration (ppm) of the DPPH solution in the different samples was calculated spectrophotometrically based on a calibration curve that was determined by linear regression:

y = 0.0284[DPPH] − 0.011,   R^2^ = 0.9997 (1)

The inhibition percentage of DPPH of each sample at the steady state was determined according to the following equation:

I (%) = [(Abs blank − Abs sample)/Abs blank] × 100(2)

### 2.7. Statistical Analysis

An analysis of variance (ANOVA) with Tukey’s test was used to initially determine any significant data differences between the groups of samples. This was followed by a principal component analysis (PCA) for an easier and more thorough understanding of any possible relationships between the studied samples regarding their phenolic and volatile compounds contents. Further, a cluster analysis (CA) was carried out to detect any similarities between the samples. The statistical significance was set at *p* < 0.05 and the results were processed using the software Statistica 12.5 (StatSoft, Inc., Tulsa, OK, USA).

## 3. Results and Discussion

### 3.1. Vinegar Production

The juice employed for the production of prickly pear vinegar, was first characterized. The initial sugar content was equivalent to 14.24 °Brix or 7.92 °Baumé, with 6.06 pH, a density of 1.055 g/mL and a total acidity of 0.82 g/100 mL expressed as citric acid. When the alcoholic fermentation was accomplished, the alcohol content was 5.27% (*v*/*v*) and the °Brix value was around 1°. At that moment, the sugar content was increased until 14 °Brix degrees was reached (the same as the juice initial °Brix value) in order to obtain a greater final alcohol content. The final alcohol content of prickly pear wine was 8.7% (*v*/*v*).

The acetic acid bacteria (AAB) strains that had presented thermo-ethanol tolerance characteristics in a previous study [32] were selected to be used as the starter culture for the acetic fermentation of the prickly pear wine at two different temperatures: 30 °C and 37 °C. The wine at 30 °C was fermented for 2 months, whereas the one at 37 °C was fermented for three months. The prickly pear vinegar produced by the surface culture at 30 °C reached a higher acidity value compared to that reached by the vinegar produced at 37 °C. This was probably due to the fact that at 30 °C all the bacteria from the *Acetobacter* genus had the capacity to produce acetic acid, which resulted in a mean value of 5.87 g/100 mL. These bacteria produced vinegars with a lower acidity compared to that of the vinegars that had been elaborated using bacteria from the *Gluconobacter* genus or with the mixture of bacteria obtained from Sherry vinegar (7.46 g/100 mL and 7.56 g/100 mL, respectively). Contrariwise, at 37 °C none of these bacteria strains or combinations of strains produced high concentrations of acetic acid, so that the *acetobacter* strains, the *gluconobacter* strains and the mixed strains obtained from Sherry vinegar achieved similar concentrations of just 1.88, 1.90 and 1.89 g/100 mL, respectively. Several reasons could explain this lower productivity, such as the effect of the high temperature on the viability of the acetic acid bacteria even if the two genera displayed thermotolerant characteristics in a previous study [32]. Other reasons could be the greater evaporation of ethanol during the fermentation at the highest temperature or a poor tolerance to a large ethanol concentration in the wine (8.7%) when at a high temperature. These are considered to be stressful conditions for acetic bacteria growth [15]. Saeki et al. demonstrated that bacterial growth at 37 °C seemed to be almost impossible and that ethanol consumption was largely delayed. In fact, the acetic bacteria did not grow at 39 °C in a medium containing over 3% ethanol [38]. Perumpuli et al. indicated that in the static fermentation processes, the lower oxygen availability in the liquid culture could negatively affect the production of acetic acids [39].

### 3.2. Volatile Compounds

In the present study, a total of 85 individual volatile compounds from different families have been determined and identified in different prickly pear samples (juice, wine and vinegars) by Stir Bar Sorptive Extraction coupled to Gas Chromatography–Mass Spectrometry (SBSE/GC-MS). In order to determine any statistical differences between the prickly pear samples regarding their composition of volatile compounds, the data were submitted to analysis of variance (ANOVA, *p* < 0.05).

Table 1 indicates the mean relative areas of all the studied compounds in the three studied matrices as well as the results from the ANOVA. ANOVA indicates if the variable under study (matrix) has a significant effect on the concentration of the studied compounds, but this statistical analysis does not indicate neither if there are differences in the concentrations of the compounds for all the levels of the studied variable, nor which level presents the higher or lower value. To obtain that information, a post hoc test such as the Tukey test was employed. This test compares all possible pairs of means and indicates which ones are different compared with each other. These results are also presented in the table by showing different letters in the same row for each analyzed compound.

Some of the volatile compounds identified in this research had been previously identified in other fruit vinegars such as lemon vinegar [33], orange vinegar [40], tomato vinegar [41], strawberry vinegar or persimmon vinegar [42], among others.

From a general point of view, almost all the volatile compounds showed significant differences according to the matrix (*p* < 0.05). This was an expected result because during the vinegar production, two different fermentation processes take place (alcoholic and acetic fermentation), and many compounds are produced and modified other than ethanol and acetic acid [15]. Some of the compounds, such as certain alcohols and esters such as 2-methyl-1-butanol, 3-methyl-1-butanol, 2-methyl-1-propanol, isobutyl acetate or hexanoic acid ethyl ester, among others, increased significantly during the alcoholic fermentation and when the acetic fermentation was accomplished, they decreased. These results are in accordance with those reported by previous research works [33,40,41]. On the other hand, other compounds such as nonenal, 2-octanone, acetic acid pentyl ester, E-3-hexenyl acetate, 2 octenal and 2,4-decadienal among others, disappeared during the transformation process from prickly pear juice into prickly pear vinegar. It should also be noted that many compounds are normally degraded during the acetic fermentation process that produces vinegar [15]. It was found that some volatile compounds such as hexanal, 1-pentanol, hexyl acetate or β-citronellol were the majority compounds in prickly pear juice, which would explain them being detected in the prickly pear vinegar, although at a lower content. Their presence in the prickly pear vinegar could also be due to the lesser degradation of compounds that takes place during acetic fermentation in surface culture processes when compared to the submerged culture method [15]. On the other hand, some of the compounds that were not detected in prickly pear juice were formed during the acetic fermentation. Some of them were *trans*-linalol oxide, dihydromethyl jasmonate, benzenepropanol, butanedioic acid diethyl ester, octanoic acid ethyl ester and benzenepropanoic acid ethyl ester, among others. Other authors who studied the production of strawberry or persimmon vinegar also found an increase in certain volatile compounds resulting from the influence of the microorganisms employed in the fermentation process [42]. Furthermore, the production of certain volatile compounds could be favored by the employment of inoculated yeasts for the alcoholic fermentation, instead of native yeasts [43]. The results obtained in this study on the volatile composition of prickly pear juice were in agreement with those reported by Arena et al. [20]. These researchers found that the major class of volatile compounds in prickly pear fruit were the alcohols represented by *trans*-2-hexen-1-ol and n-hexanol, even though numerous esters and carbonyl compounds were also present at low concentrations. No data on prickly pear wine or vinegar regarding their volatile composition have been found in the literature.

**Figure 1:**
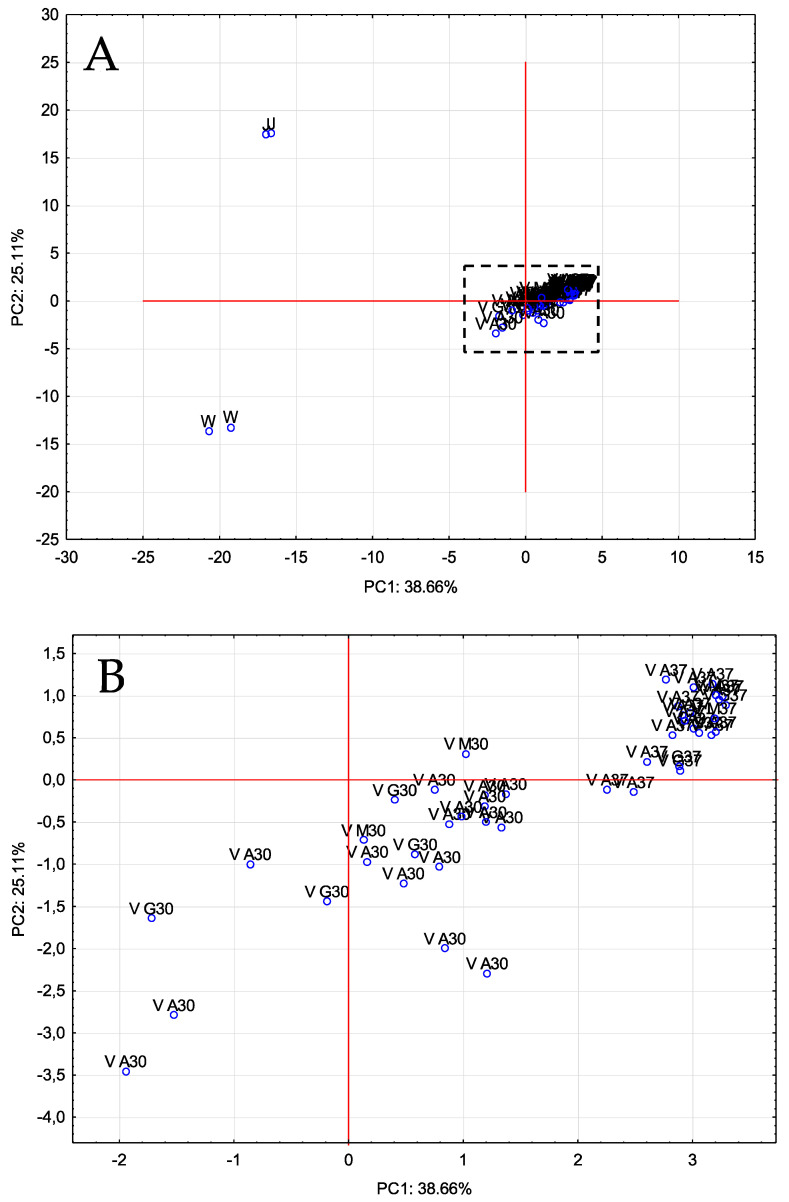
PCA of volatile compounds. Distribution of all samples onto the plane defined by the first two PCs. (**A**): original graph; (**B**): zoomed area. J: prickly pear juice; W: prickly pear wine; V: prickly pear vinegar.

In order to corroborate the differences that took place during the vinegar production process, the data obtained were submitted to a multivariate statistical study (principal component analysis—PCA). This analysis allowed us to identify eight PCs that could explain 94.61% of the total variability (eigenvalues > 1). Figure 1 represents the distribution of the samples on the plane defined by the first two PCs.

As can be seen, PC1 allowed to distinguish vinegar samples (V) from juice (J) and wine (W), whereas PC2 was able to separate the three prickly pear matrixes. The volatile compounds that contributed more and with a greater influence on PC1 were as follows: methyl salicylate, 1-hexanol, 1-heptanol, 2-octanone, 1-pentanol, *trans*-2-*cis*-6-nonadienal and 3-hexen-1-ol (E). Regarding to PC2, these ones were: 3-hexen-1-ol acetate (Z), *trans*-2-decenol, *trans, cis*-2,6-nonadien-1-ol, 2-octenoic acid, 2-hexen-1-ol acetate (E), 2-hexen-1-ol (E) and nonenal.

To study the effect from certain parameters, such as fermentation temperature and acetic acid bacteria on the volatile composition of the prickly pear vinegar, the prickly pear wine was inoculated with a different genus of acetic acid bacteria (*Acetobacter*, *Gluconobacter* and a mixture of bacteria from Sherry vinegar) and incubated at 30 °C and 37 °C. Table 2 shows the results from the analysis of variance considering temperature and genus as the independent variables and volatile compounds as the dependent ones. As can be seen, most of the volatile compounds were significantly affected by fermentation temperature, whereas no significant differences between the volatile composition of the vinegars produced by the different bacteria inoculum were detected. Hence, the type of acetic acid bacteria inoculated does not seem to be relevant with respect to the volatile composition of the vinegars. Other studies have reported slight differences in the taste of vinegars produced using *Gluconobacter* genus bacteria when compared to those produced using *Acetobacter*, since the former ones generate greater amounts of gluconate [44]. Kim et al. observed that the production of certain metabolites in tomato vinegar depended on the strain of acetic acid bacteria used and that the fermentation temperature also had a significant influence on the production of such metabolites [45].

Table 3 indicates the mean relative areas of the volatile compounds in the vinegars produced at 30 °C and at 37 °C. It can be observed that most of the volatile compounds reached higher concentration values in the vinegar that had been produced at 30 °C compared to those corresponding to the vinegar produced at 37 °C. According to Liu et al., the aroma compounds and their concentrations were influenced by the fermentation temperature, so that as the temperature was increased, the number of volatiles would decrease [46]. Consequently, certain compounds, such as 1,3-dioxolane, 2,4,5-trimethyl-, 1-hexanol, 3-hexen-1-ol (E), 3-hexen-1-ol (Z), cyclopentene or *trans*-2-*cis*-6-nonadienal, would even disappear when the acetic fermentation was conducted at 37 °C. These changes in the concentration of certain compounds could be explained by their possible reduction by evaporation when submitted to high temperatures (37 °C), which, in some cases, might lead to their total disappearance [15]. For example, cyclopentene and 1,3-dioxolane have boiling points at atmospheric pressure of 44 °C and 74 °C, respectively; however, the mean relative area detected for some compounds, such as *trans*-linalooloxide, *cis*-linalooloxide, 1-hexanol 2-ethyl-, benzaldehyde, 1-nonanol and dihydromethyl jasmonate among others was greater at 37 °C than at 30 °C. This was probably due to changes in the metabolism of the bacteria as the temperature varied. Thus, the production of 2,3-butanediol and butanoic acid during the elaboration of tomato vinegar at different temperatures was studied by Kim et al. [45] and 2,3-butanediol was detected at greater concentrations in those vinegars produced at a higher temperature (34 °C), whereas butanoic acid was only found in the vinegars produced at 30 °C. In the present study, similar behavior by both of these compounds was observed. Although the increment of the fermentation temperature led, as expected, to a general reduction in the volatile content in the vinegars, partial fermentations were accomplished at 37 °C even if the optimal growth temperature for acetic acid bacteria is between 25 and 30 °C [15].

**Figure 2:**
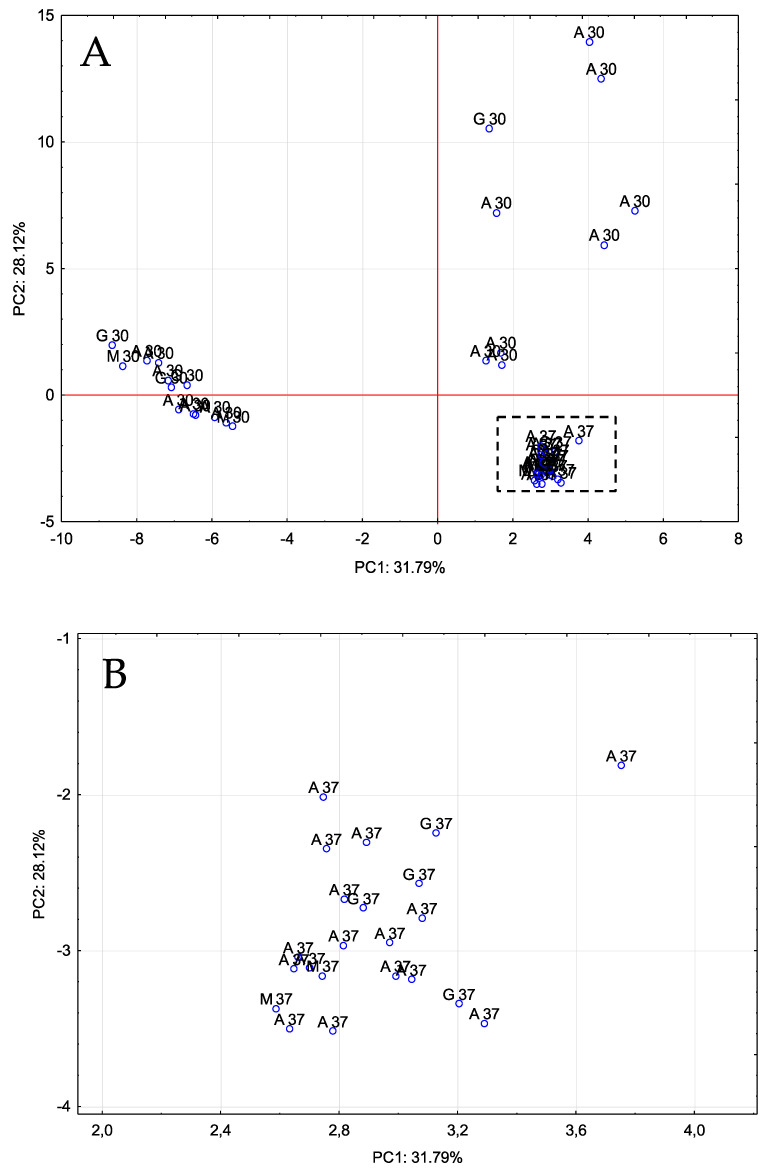
PCA on volatile compounds. Distribution of all vinegar samples onto the plane defined by the first two PCs. (**A**): original graph; (**B**): zoomed area. A 30/A 37: *Acetobacter* at 30 °C and 37 °C, G 30/G 37: *Gluconobacter* at 30 °C and 37 °C, M 30/M 37: Mixture of bacteria at 30 °C and 37 °C.

The resulting data were submitted to a multivariate statistical study (principal component analysis, PCA), which allowed us to identify 11 PCs that explained 92.7% of the total variability (eigenvalues > 1). Figure 2 shows the distribution of all the vinegar samples on the plane defined by PC1 and PC2. As it can be seen, PC1 separated the vinegars produced at 37 °C from some of the vinegars fermented at 30 °C, which were mainly those vinegars that had been fermented using *gluconobacter*. PC2, on the other hand, was able to separate all the vinegars fermented at 37 °C from those fermented at 30 °C. PC1 was mainly related to the presence of acids, such as hexanoic acid, nonanoic acid, isovaleric acid, isobutyric acid and acetic acid, while PC2 was related to certain alcohols, such as 1-octanol, linalool, 3-methyl-1-butanol, 3-hexen-1-ol (Z) or 1-pentanol.

**Figure 3:**
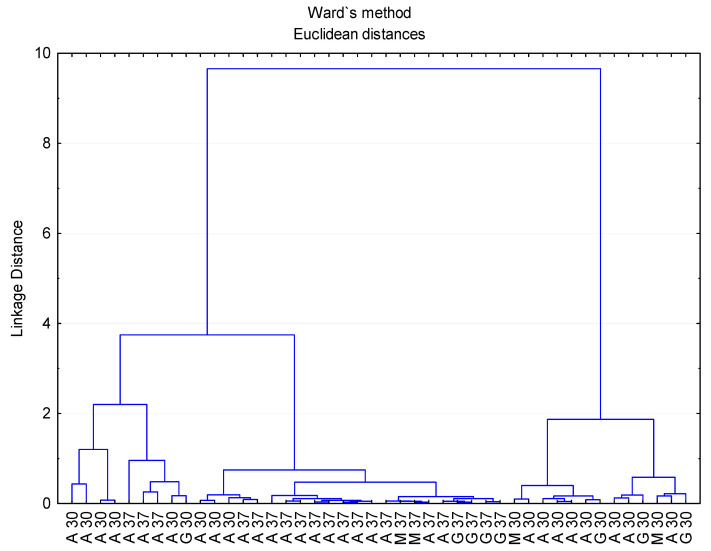
Cluster analysis taking into consideration the composition of volatile compounds of vinegar samples. A 30/A 37: *Acetobacter* at 30 °C and 37 °C, G 30/G 37: *Gluconobacter* at 30 °C and 37 °C, M 30/M 37: Mixture of bacteria at 30 °C and 37 °C.

To corroborate these results, the set of data was also submitted to a hierarchical agglomerative cluster analysis (Figure 3). The squared Euclidean distance as metric and the Ward method as the amalgamation rule were employed to set up the clusters. As illustrated, three groups were obtained: vinegars produced by *acetobacter* at 30 °C and 37 °C (A 30 and A 37), vinegars produced by the three types of bacteria inoculum at 37 °C (A/G/M 37) and vinegars produced by the three types of bacteria inoculum at 30 °C (A/G/M 30). These groupings revealed that the influence that the acetification temperature exerted on the vinegars’ volatile profiles was quite high and even more significant than that corresponding to the particular acetic acid bacteria inocula employed.

### 3.3. Phenolic Compounds and Antioxidant Activity

#### 3.3.1. Phenolic Composition

In the present study, a total of 16 polyphenolic compounds in prickly pear samples (juice, wine and vinegar) have been studied. To determine the statistical differences between prickly pear samples regarding their polyphenolic compounds content, the data were submitted to analysis of variance (ANOVA, *p* < 0.05). Table 4 displays the mean concentrations of the different polyphenolic compounds depending on the matrix. 

Some of the compounds among those identified in prickly pear samples were hydroxycinnamic and benzoic acids, such as ferulic acid, gallic acid, caffeic acid and cinnamic acid as well as and flavanones, such as hesperidin and naringin. These compounds had been previously identified as the major compounds in orange samples (juice, wine and vinegar) [40]. Seven compounds presented similar concentrations in the three studied matrices, such as gallic acid, hydroxy-tyrosol, tyrosol or ferulic acid. The rest of the phenolic compounds studied showed significantly different contents when the matrix was transformed from prickly pear juice into prickly pear vinegar. A few compounds (epigallocatechin, syringic acid, protocatechualdehyde and cinnamic acid) increased their concentrations gradually during the elaboration process of the vinegar. On the other hand, just two compounds (ethyl gallate and *p*-hydroxybenzoic acid) decreased their concentration as the juice was transformed into vinegar. Other authors have also reported decreases of phenolic compounds over the production of pomegranate vinegars [47,48]. In our case, hesperidin’s concentration decreased significantly during the alcoholic fermentation and later on increased when the acetic fermentation was accomplished, whereas *m*-coumaric acid disappeared as the transformation process from prickly pear juice into prickly pear vinegar took place. Caffeic acid and vanillic acid were not detected in prickly pear juice or wine, but they were present after the acetic fermentation process, and epigallocatechin and cinnamic acid were not detected in the juice stage, but were identified in the following stages. A possible hypothesis could be that these compounds were produced and/or released during the fermentation processes. In fact, it has been previously reported that by the selection of the bacteria strain employed in the fermentation process, some bioactive components could be promoted to the final product [15]. Tyrosol was the main compound found in all the samples and it was actually detected at a high concentration in juice, which would explain its presence in the vinegar. This is not in agreement with the results from certain previous studies on other fruit vinegars, where an increase in tyrosol content after the alcoholic fermentation was reported [33,41]. With regard to gallic acid, a previous study reported that it was the main phenolic compound at variable concentrations in a number of varieties of prickly pear juice, except for the juice obtained from Tapona fruit, where no gallic was detected. Syringic acid was the second most abundant phenolic compound in the juices, although it was not identified in the juice from two of the studied varieties [49]. No other investigations concerning the phenolic composition of prickly pear wine or vinegar have been found in the literature.

**Figure 4:**
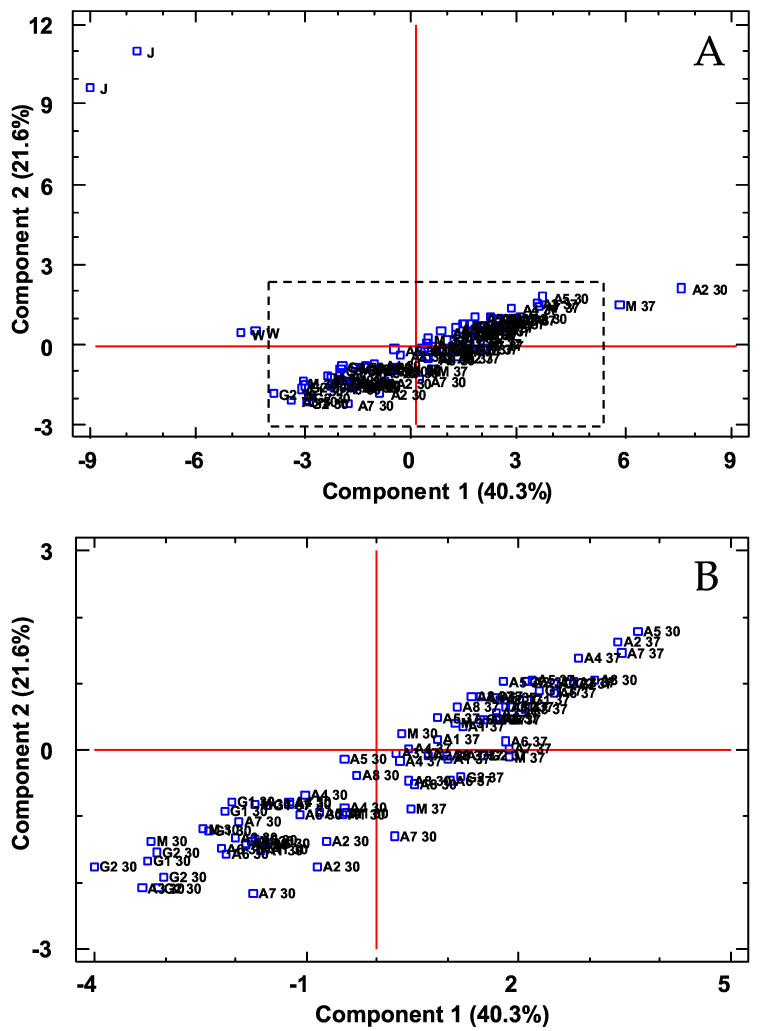
PCA on phenolic compounds. Distribution of all the samples onto the plane defined by the first two PCs. (**A**): original graph; (**B**): zoomed area. J: prickly pear juice; W: prickly pear wine; A/G/M: prickly pear vinegar.

In order to gain further insight into the evolution of the process, the polyphenolic data obtained were also submitted to multivariate statistical study (principal component analysis, PCA and cluster analysis, CA). Thus, four PCs were detected that were able to explain 78.92% of the total variability according to the Kaiser criterion (eigenvalues > 1). By attending to just the first two components, 61.99% of the variability could be explained. Figure 4 shows the plot, including the samples on the plane defined by PC1 and PC2. 

**Figure 5:**
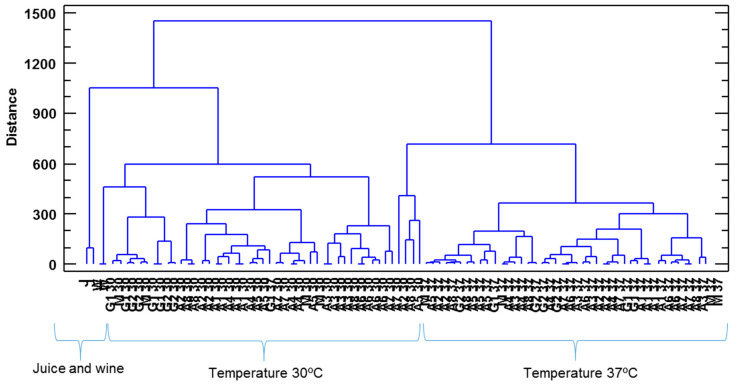
Cluster analysis taking into consideration the composition of polyphenolic compounds of the three prickly pear matrixes. J: prickly pear juice; W: prickly pear wine; A/G/M: prickly pear vinegar.

As can be seen, PC1 was able to separate the three prickly pear matrices, whereas PC2 separated the juice samples from the wine and vinegar ones. The polyphenolic compounds that contributed more and with a greater influence on PC1 were as follows: epigallocatechin, tyrosol, syringic acid, protocatechualdehyde and caffeic acid, whereas PC2 was affected mainly by ethyl gallate, *m*-coumaric acid, hesperidin and *p*-hydroxybenzoic acid. Regarding the CA, the squared Euclidean distance as metric and the Ward method as the amalgamation rule were employed to set up the clusters. As can be seen (Figure 5), three groups were obtained as follows: juice and wine; vinegars produced at 30 °C and vinegars produced at 37 °C. 

Table 5 shows the results from the Analysis of Variance on the polyphenolic composition of prickly pear vinegars according to temperature and bacteria inoculum differences. As can be seen, the concentration of polyphenolic compounds was significantly affected by both fermentation temperature and acetic acid bacteria inoculum. Except for gallic and vanillic acid, the concentration levels of all the polyphenolic compounds identified in prickly pear vinegars were affected by fermentation temperature changes, so that temperature was confirmed as the most important variable. Similar results, regarding other analytes, had been obtained by Kim et al. when elaborating tomato vinegar [45].

Table 6 includes the mean concentrations of the polyphenolic composition of the vinegars produced at 30 °C and at 37 °C and using the three types of bacteria inocula. It can be seen that contrarily to the behavior exhibited by their volatile composition, the vinegar that had been produced at 37 °C presented significantly higher concentrations of most phenolic compounds, except for gallic acid, hydroxy-tyrosol, vanillic acid, quercetin, cinnamic acid and p-hydroxybenzoic acid. It could, therefore, be said that, in terms of polyphenolic composition and considering the beneficial antioxidant effect from polyphenols reported by other studies on fruit vinegars, the vinegars produced at higher temperatures might be healthier than those produced at 30 °C [15]. Regarding the type of bacteria inoculum employed, no clear trend has been revealed in terms of a higher or lower concentration of polyphenolic compounds associated with the particular bacteria inoculum used to produce the vinegars (Table 6).

**Figure 6:**
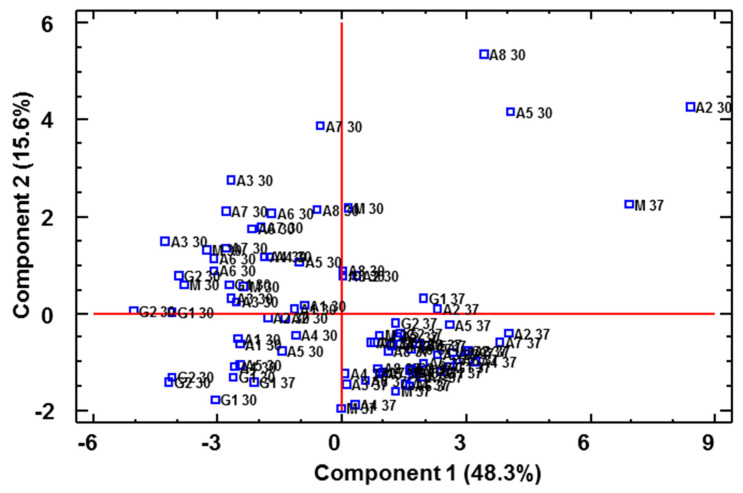
PCA on phenolic compounds. Distribution of all the vinegar samples onto the plane defined by the first two PCs. A 30/A 37: *Acetobacter* at 30 °C and 37 °C, G 30/G 37: *Gluconobacter* at 30 °C and 37 °C, M 30/M 37: Mixture of bacteria at 30 °C and 37 °C.

These data on vinegar polyphenol content were submitted to a multivariate statistical study (PCA) and three PCs were identified that were able to explain 73.28% of the total variability (eigenvalues > 1). Figure 6 illustrates which of the vinegar samples were located on the plane defined by PC1 and PC2. Thus, it could be observed that these two components were capable of separating all the vinegars produced at 30 °C from those fermented at 37 °C, with 37 °C-elaborated vinegars located at the positive values of PC1, whereas the 30 °C-elaborated vinegars were located at the negative value area of the same PC. The compounds that contributed the most to the first principal component (PC1) were epigallocatechin, tyrosol and protocatechualdehyde, while for PC2, gallic acid, hydroxy-tyrosol, vanillic acid and p-hydroxybenzoic acid.

#### 3.3.2. Antioxidant Activity

The antioxidant activity of prickly pear samples (juice, wine and vinegars) was determined by means of DPPH radical scavenging (expressed as EC_20_). This is a fast, simple, economical and widely used method to measure the overall antioxidant capacity and the free radical scavenging activity of fruits and vegetable juices. This method is carried out in a mixture methanol/water, which facilitates the extraction of antioxidant compounds from the sample. Moreover, DPPH is allowed to react with the whole sample and sufficient time given in the method allows DPPH to react slowly even with weak antioxidants; however, this method is limited because DPPH radical interacts with other radicals and some problems of linearity can be found with different ratios of antioxidant/DPPH. In addition, DPPH is sensitive to some Lewis bases and solvent types, as well as oxygen, and it can only be soluble in organic solvents. Another drawback is that the absorbance of DPPH in methanol and acetone decreases under light [50]. In spite of these drawbacks, this methodology is nowadays commonly employed thanks to the above-mentioned advantages.

Depending on the matrix, the results revealed that there was a clear difference with respect to the antioxidant activity corresponding to juice, wine or vinegars (0.618 ± 0.000 mg/mL, 0.456 ± 0.035 mg/mL, 1.459 ± 0.055 mg/mL, respectively). Although there is no clear evidence that in vitro antioxidant activity values have relation to a biological significance after the consumption of any food, it could provide an initial idea about their possible healthy character. The DPPH antioxidant scavenging capacity of prickly pear juice from Moroccan *O. ficus indica* in our study was similar to that registered for Tunisian *O. ficus indica* pulp [51]. As can be seen, the highest antiradical activity was exhibited by the prickly pear wine with the lowest EC_20_ values, which might be explained by the solubility of the polymerized polyphenols in ethanol, that resulted in antioxidant compounds concentration increments as the content of ethanol increased over the alcoholic fermentation. The results obtained in our study are in contradiction with those obtained by Kongkiattikajorn [52], who reported that the total antioxidant activity of Roselle vinegar was significantly higher than that of Roselle juice and wine because of a greater number of anthocyanins in the vinegar in relation to that found in the wine and juice. A previous study on the evolution of the antioxidant capacity of fermenting persimmon juice determined by DPPH assays, showed that the antioxidant capacity went up during the alcoholic fermentation and acetification, which is in line with the presence of a greater amount of flavan-3-ols and condensed tannins [53].

Regarding the antioxidant activity of vinegars produced by inoculating different bacteria genus, the data revealed that the vinegars produced by the bacteria mixture from Sherry vinegar displayed a greater activity (0.833 ± 0.0440 mg/mL) than those produced by means of either *Gluconobacter* (1.018 ± 0.030 mg/mL) or *Acetobacter* genus (1.636 ± 0.061 mg/mL). This may be explained by a combination of a greater amount of certain bioactive compounds such as hydroxy-tyrosol or *p*-hydroxybenzoic acid in the vinegar produced by the mixture of bacteria found in Sherry vinegar lees, compared to those obtained by *Gluconobacter* and *Acetobacter*, in decreasing order (Table 6). These two phenolic compounds have been previously related to the high antioxidant activity of other vegetal matrices such as *Jasminum* species [54]. In addition, hydroxy-tyrosol is also considered the main responsible for the bioactivity of olives and olive pits [55].

## 4. Conclusions

Prickly pear vinegar has been produced by the surface culture at different temperatures and with different AAB inocula. The yields from fermentations at 30 °C ranged between 67.47% and 86.89%, whereas the yields at 37 °C did not reach over 21.83% in any case. Eighty-five separate volatile compounds from different families and sixteen polyphenolic compounds have been identified in the vinegars. By observing the effect of the acetification temperature on volatile compounds, it was concluded that at the highest temperature tested (37 °C), the concentration of these compounds decreased; however, the highest content of polyphenolic compounds was detected when the vinegars were produced at 37 °C. Further, a greater antioxidant activity was exhibited by prickly pear wine than by juice or vinegars. The vinegar produced by a mixture of AAB from Sherry vinegar displayed the highest antiradical activity compared to those corresponding to the vinegars elaborated using other AAB inocula. It can be concluded that changes in the temperature levels during the fermentation of prickly pear to produce vinegar can have a significant impact on the polyphenolic and volatile composition of the final vinegars, and although their volatile content decreases when fermented at higher temperature, partial fermentations could be carried out at 37 °C as long as thermotolerant bacteria are employed, so that the final vinegar would be richer in polyphenols.

## Figures and Tables

**Table 1:** Retention times (RT), mean relative areas and standard deviations (SD) of volatile compounds identified by SBSE-GC-MS in different prickly pear matrices (juice, wine, vinegar). Results of analysis of variance taking into account the matrix.

Compounds	RT	Juice	Wine	Vinegar	ANOVA	
		Mean ± SD	Mean ± SD	Mean ± SD	F Ratio	*p*-Value
Ethyl acetate	8.89	0.3421 ± 0.0428 a	13.96 ± 1.169 b	0.1669 ± 0.2122 a	3126	0.0000 *
1,3-Dioxolane, 2,4,5-trimethyl-	11.72	ND a	0.1049 ± 0.0517 b	0.0089 ± 0.0256 a	13.5	0.0000 *
Diacetyl	13.08	0.0055 ± 0.0003	ND	0.0093 ± 0.0130	0.590	0.5561
Isobutyl acetate	15.00	0.0058 ± 0.0002 a	0.0915 ± 0.0004 b	0.0047 ± 0.0105 a	68.4	0.0000 *
Hexanal	18.01	0.0110 ± 0.0001 b	0.0028 ± 0.0002 a	0.0026 ± 0.0011 a	56.7	0.0000 *
2-methyl-1-propanol	19.04	0.0022 ± 0.0004 a	0.0334 ± 0.0003 b	0.0027 ± 0.0062 a	24.2	0.0000 *
Isoamyl acetate	19.74	0.0555 ± 0.0161 a	0.8122 ± 0.0257 b	0.0402 ± 0.0756 a	104	0.0000 *
Acetic acid, pentyl ester	21.59	0.0321 ± 0.0017 c	0.0093 ± 0.0003 b	ND a	30,820	0.0000 *
2,6-dimethyl-4-heptanone	21.69	0.0062 ± 0.0005	0.0086 ± 0.0011	0.0045 ± 0.0047	0.863	0.4254
2-methyl-1-butanol	23.11	0.0032 ± 0.0025 a	0.4001 ± 0.0296 b	0.0234 ± 0.0442 a	72.6	0.0000 *
3-methyl-1-butanol	23.24	0.0050 ± 0.0005 a	0.3833 ± 0.0314 b	0.0279 ± 0.0473 a	56.6	0.0000 *
Furan, 2-pentyl-	23.67	0.0068 ± 0.0009 b	ND a	ND a	4884	0.0000 *
Hexanoic acid, ethyl ester	23.85	0.0115 ± 0.0017 a	0.0287 ± 0.0257 b	0.0035 ± 0.0042 a	27.6	0.0000 *
Styrene	24.50	0.0015 ± 0.0002 b	0.0075 ± 0.0031 c	0.0003 ± 0.0002 a	331	0.0000 *
1-pentanol	24.58	0.0039 ± 0.0001 c	0.0025 ± 0.0002 b	0.0001 ± 0.0003 a	220	0.0000 *
Hexyl acetate	25.50	0.0460 ± 0.0055 c	0.0108 ± 0.0027 b	0.0012 ± 0.0028 a	255	0.0000 *
Acetoin	25.72	ND	ND	0.1369 ± 0.1829	1.09	0.3384
Acetol	26.08	0.0079 ± 0.0008 a	0.0489 ± 0.0150 b	0.0085 ± 0.0068 a	33.2	0.0000 *
2-octanone	26.12	0.0068 ± 0.0005 b	0.0132 ± 0.0031 c	ND a	1904	0.0000 *
3-Hexen-1-ol, acetate, (Z)	26.90	0.0054 ± 0.0000 b	ND a	ND a	1.30 × 10^9^	0.0000 *
E-3-hexenyl acetate	26.91	0.0054 ± 0.0001 c	0.0028 ± 0.0004 b	ND a	15,409	0.0000 *
2-Hexen-1-ol, acetate, (E)	27.45	0.0304 ± 0.0032 b	ND a	ND a	7647	0.0000 *
Ethyl lactate	27.55	0.0005 ± 0.0000 a	0.2038 ± 0.0148 ab	0.3701 ± 0.1801 b	4.98	0.0089 *
1-hexanol	28.14	0.0288 ± 0.0009 b	0.0334 ± 0.0028 b	0.0006 ± 0.0019 a	473	0.0000 *
3-Hexen-1-ol, (E)-	28.41	0.0033 ± 0.0005 b	0.0046 ± 0.0021 c	0.0001 ± 0.0005 a	96.4	0.0000 *
3-Hexen-1-ol, (Z)-	29.20	0.0025 ± 0.0003 b	0.0022 ± 0.0001 b	0.0002 ± 0.0005 a	34.4	0.0000 *
2-Hexen-1-ol, (E)-	29.89	0.0072 ± 0.0007 b	ND a	ND a	8389	0.0000 *
Acetic acid	30.79	0.0444 ± 0.0003	0.0503 ± 0.0043	0.2439 ± 0.2641	1.08	0.3428
2-octenal	31.31	0.0268 ± 0.0040 b	ND a	ND a	3772	0.0000 *
Octanoic acid, ethyl ester	31.46	ND a	0.1203 ± 0.0103 b	0.0007 ± 0.0031 a	1306	0.0000 *
*trans*-linalooloxide	31.60	ND	ND	0.0079 ± 0.0054	4.20	0.0180 *
1-heptanol	31.82	0.0033 ± 0.0001 b	0.0056 ± 0.0008 c	ND a	5231	0.0000 *
2,4-heptadienal, (E,E)-	32.29	0.0037 ± 0.0010 b	ND a	ND a	1129	0.0000 *
*cis*-linalooloxide	32.61	0.0003 ± 0.0000 a	0.0013 ± 0.0005 b	0.0056 ± 0.0015 c	20.5	0.0000 *
4-Octenoic acid, ethyl ester, (Z)-	32.73	ND a	0.0041 ± 0.0001 b	ND a	189,066	0.0000 *
1-Hexanol, 2-ethyl-	33.05	0.0016 ± 0.0001	0.0097 ± 0.0009	0.0160 ± 0.0139	1.25	0.2914
Cyclopentene	34.17	0.0012 ± 0.0001 b	0.0011 ± 0.0001 b	0.0001 ± 0.0002 a	34.2	0.0000 *
Benzaldehyde	34.41	0.0012 ± 0.0001 a	0.0015 ± 0.0001 a	0.0118 ± 0.0064 b	5.17	0.0075 *
2,3-butanediol	34.67	0.0010 ± 0.0002 a	0.0059 ± 0.0008 ab	0.0151 ± 0.0069 b	5.73	0.0046 *
Linalool	35.02	0.0190 ± 0.0007 b	0.0673 ± 0.0007 c	0.0012 ± 0.0017 a	1671	0.0000 *
Nonenal	35.14	0.0075 ± 0.0008 b	ND a	ND a	6850	0.0000 *
Isobutyric acid	35.40	0.0047 ± 0.0006	0.0221 ± 0.0012	0.0164 ± 0.0157	0.705	0.4969
1-octanol	35.53	0.0079 ± 0.0006 b	0.0339 ± 0.0005 c	0.0010 ± 0.0022 a	222	0.0000 *
*trans*-2-*cis*-6-nonadienal	36.82	0.0117 ± 0.0008 b	0.0314 ± 0.0023 c	0.0001 ± 0.0004 a	4988	0.0000 *
*trans*-2-Decenol	37.43	0.0066 ± 0.0001 b	ND a	ND a	1,139,269	0.0000 *
Butanoic acid	37.65	ND	ND	0.0011 ± 0.0014	1.17	0.3135
Sulfide, allyl methyl	38.12	0.0022 ± 0.0001 a	0.0115 ± 0.0005 c	0.0043 ± 0.0027 b	8.08	0.0006 *
Decanoic acid, ethyl ester	38.82	ND a	0.0248 ± 0.0022 b	ND a	10,625	0.0000 *
Isovaleric acid	39.17	ND	ND	0.0341 ± 0.0390	1.50	0.2288
1-nonanol	39.23	0.0180 ± 0.0004 b	0.1010 ± 0.0064 c	0.0039 ± 0.0041 a	557	0.0000 *
Butanedioic acid, diethyl ester	39.62	ND a	0.0166 ± 0.0007 ab	0.0259 ± 0.0099 b	7.60	0.0009 *
*trans, cis*-2,6-nonadienyl acetate	40.23	0.0220 ± 0.0030 b	ND a	ND a	4479	0.0000 *
α-Terpineol	40.76	0.0048 ± 0.0001 a	0.0235 ± 0.0005 b	0.0083 ± 0.0044 a	12.7	0.0000 *
2-Nonen-1-ol, (E)-	41.13	0.0518 ± 0.0029 c	ND a	0.0017 ± 0.0044 b	128	0.0000 *
*cis*-6-nonenol	41.22	0.0394 ± 0.0026 a	0.2145 ± 0.0073 b	0.0221 ± 0.0291 a	44.0	0.0000 *
Benzyl acetate	41.59	0.0131 ± 0.0042	0.0046 ± 0.0002	0.0057 ± 0.0054	1.91	0.1536
β-Citronellol	42.96	0.1333 ± 0.0019 c	0.0239 ± 0.0014 b	0.0028 ± 0.0014 a	8759	0.0000 *
*trans, cis*-2,6-Nonadien-1-ol	42.96	0.1340 ± 0.0011 b	ND a	ND a	1,226,155	0.0000 *
Methyl salicylate	43.47	0.0196 ± 0.0012 b	0.0256 ± 0.0002 c	0.0011 ± 0.0011 a	711	0.0000 *
Ethyl phenylacetate	43.60	0.0027 ± 0.0000	0.0223 ± 0.0003	0.0145 ± 0.0189	0.568	0.5687
Phenethyl acetate	44.78	0.0383 ± 0.0049	0.1995 ± 0.0011	0.2040 ± 0.2131	0.605	0.5481
2,4-decadienal	44.98	0.0065 ± 0.0019 b	ND a	ND a	1056	0.0000 *
β-damascenone	45.24	ND a	0.0075 ± 0.0048 b	ND a	206	0.0000 *
Hexanoic acid	45.41	0.0048 ± 0.0002	0.0119 ± 0.0012	0.0183 ± 0.0126	1.37	0.2572
Geraniol	45.74	0.0067 ± 0.0004 b	0.0167 ± 0.0001 c	0.0013 ± 0.0021 a	58.0	0.0000 *
*cis*-geranylacetone	46.28	0.0018 ± 0.0002 a	0.0129 ± 0.0012 b	0.0026 ± 0.0037 a	7.63	0.0009 *
Benzyl alcohol	46.58	0.0043 ± 0.0001 a	0.0072 ± 0.0001 a	0.0110 ± 0.0023 b	11.3	0.0000 *
Benzenepropanoic acid, ethyl ester	47.18	ND a	0.0420 ± 0.0018 b	0.0054 ± 0.0062 a	36.0	0.0000 *
Phenylethyl alcohol	47.88	0.0096 ± 0.0011 a	0.1723 ± 0.0041 ab	0.2644 ± 0.1385 b	3.77	0.0267 *
3-Phenyl-1-propanol, acetate	49.09	0.0038 ± 0.0015	0.0092 ± 0.0008	0.0082 ± 0.0155	0.084	0.9194
2,4-Decadien-1-ol	50.65	0.0030 ± 0.0004 b	ND a	ND a	5293	0.0000 *
Phenol	50.73	0.0012 ± 0.0004 a	0.0045 ± 0.0008 b	0.0035 ± 0.0008 b	8.41	0.0005 *
4-hydroxynonanoic acid lactone	52.19	0.0225 ± 0.0014 a	0.0315 ± 0.0003 a	0.0521 ± 0.0073 b	23.6	0.0000 *
Benzenepropanol	52.31	ND a	0.0046 ± 0.0004 ab	0.0050 ± 0.0023 b	4.63	0.0122 *
Octanoic acid	52.57	0.0275 ± 0.0023 a	0.1683 ± 0.0031 b	0.1106 ± 0.0525 ab	3.78	0.0264 *
Ethyl cinnamate	55.18	ND a	0.0135 ± 0.0000 b	0.0031 ± 0.0019 a	34.1	0.0000 *
Cinnamyl acetate	55.67	0.0030 ± 0.0007 b	0.0010 ± 0.0001 a	0.0004 ± 0.0003 a	58.3	0.0000 *
Nonanoic acid	55.92	0.0136 ± 0.0003 a	0.0817 ± 0.0008 b	0.0432 ± 0.0269 ab	3.34	0.0398 *
Thymol	56.40	0.0027 ± 0.0001 a	0.0146 ± 0.0003 b	0.0045 ± 0.0013 a	59.4	0.0000 *
2-Octenoic acid	56.43	0.0042 ± 0.0004 b	ND a	ND a	10,244	0.0000 *
Decanoic acid	59.15	0.0253 ± 0.0118 a	0.1164 ± 0.0007 b	0.0256 ± 0.0176 a	26.4	0.0000 *
2-nonenoic acid	59.62	0.0089 ± 0.0007 b	0.0016 ± 0.0004 a	0.0024 ± 0.0016 a	17.2	0.0000 *
Dihydromethyl jasmonate	59.95	ND a	ND a	0.0013 ± 0.0010 b	3.35	0.0396 *
Ɣ-dodecalactone	63.08	0.0136 ± 0.0031 a	0.0530 ± 0.0008 b	0.0169 ± 0.0055 a	43.4	0.0000 *
Dodecanoic acid	66.58	0.0166 ± 0.0086 a	0.1231 ± 0.0053 b	0.0082 ± 0.0089 a	165	0.0000 *
Tetradecanoic acid	78.46	0.0052 ± 0.0004 a	0.0123 ± 0.0014 b	0.0024 ± 0.0025 a	17.0	0.0000 *

Different letters in the same row indicate significant differences according to Tukey’s test (*p* < 0.05). * Values are significant at *p* < 0.05; ND: Not detected.

**Table 2:** Analysis of variance considering the effect of temperature (30 °C/37 °C) and bacteria inoculum (*Acetobacter* (A)/*Gluconobacter* (G)/Mixture of bacteria (M)) on volatile compounds of prickly pear vinegar produced by surface culture.

Compounds	Temperature (30 °C/37 °C)		Bacteria Inoculum (A/G/M)	
	F Ratio	*p*-Value	F Ratio	*p*-Value
Ethyl acetate	0.076	0.7833	0.425	0.6546
1,3-Dioxolane, 2,4,5-trimethyl	11.4	0.0011 *	0.980	0.3795
Diacetyl	35.3	0.0000 *	0.582	0.5610
Isobutyl acetate	0.057	0.8108	0.196	0.8219
Hexanal	11.4	0.0011 *	2.24	0.1126
2-methyl-1-propanol	19.1	0.0000 *	0.070	0.9317
Isoamyl acetate	0.016	0.8987	0.374	0.6885
2,6-dimethyl-4-heptanone	6.91	0.0102 *	0.281	0.7551
2-methyl-1-butanol	17.0	0.0001 *	1.39	0.2524
3-methyl-1-butanol	24.4	0.0000 *	0.486	0.6167
Hexanoic acid, ethyl ester	0.766	0.3837	0.801	0.4522
Styrene	1.10	0.2962	0.967	0.3844
1-pentanol	10.0	0.0021 *	0.926	0.4000
Hexyl acetate	13.0	0.0005 *	7.46	0.0010*
Acetoin	59.1	0.0000 *	1.16	0.3161
Acetol	0.308	0.5799	2.44	0.0927
Ethyl lactate	0.740	0.3921	2.92	0.0593
1-hexanol	8.47	0.0046 *	0.412	0.6635
3-Hexen-1-ol, (E)-	5.08	0.0267 *	0.945	0.3927
3-Hexen-1-ol, (Z)-	12.8	0.0006 *	0.622	0.5392
Acetic acid	47.6	0.0000 *	1.43	0.2443
Octanoic acid, ethyl ester	0.008	0.9286	0.833	0.4382
*trans*-linalooloxide	353	0.0000 *	0.091	0.9127
*cis*-linalooloxide	234	0.0000 *	0.100	0.9042
1-Hexanol, 2-ethyl-	4378	0.0000 *	0.045	0.9558
Cyclopentene	11.0	0.0013 *	0.578	0.5628
Benzaldehyde	24.6	0.0000 *	4.33	0.0161 *
2,3-butanediol	23.2	0.0000 *	1.40	0.2504
Linalool	45.4	0.0000 *	0.639	0.5303
Isobutyric acid	60.3	0.0000 *	1.30	0.2777
1-octanol	16.3	0.0001 *	0.907	0.4075
*trans*-2-*cis*-6-nonadienal	12.3	0.0007 *	1.07	0.3465
Butanoic acid	74.5	0.0000 *	0.959	0.3871
Sulfide, allyl methyl	3.75	0.0559	1.64	0.1992
Isovaleric acid	50.9	0.0000 *	2.05	0.1350
1-nonanol	10.7	0.0015 *	2.10	0.1279
Butanedioic acid, diethyl ester	39.1	0.0000 *	1.44	0.2407
α-terpineol	490	0.0000 *	0.490	0.6140
2-Nonen-1-ol, (E)-	4.63	0.0342 *	3.05	0.0523
*cis*-6-nonenol	6.50	0.0126 *	1.02	0.3623
Benzyl acetate	31.0	0.0000 *	2.66	0.0755
β-Citronellol	30.3	0.0000 *	2.34	0.1026
Methyl salicylate	72.0	0.0000 *	2.77	0.0682
Ethyl phenylacetate	15.8	0.0001 *	0.847	0.4322
Phenethyl acetate	34.4	0.0000 *	0.050	0.9509
Hexanoic acid	35.6	0.0000 *	2.35	0.1007
Geraniol	29.8	0.0000 *	1.95	0.1480
*cis*-geranylacetone	0.008	0.9261	0.229	0.7954
Benzyl alcohol	0.875	0.3522	3.33	0.0404 *
Benzenepropanoic acid, ethyl ester	66.9	0.0000 *	0.394	0.6754
Phenylethyl alcohol	1.14	0.2874	2.18	0.1191
3-Phenyl-1-propanol, acetate	11.1	0.0013 *	0.767	0.4676
Phenol	21.0	0.0000 *	0.559	0.5735
4-hydroxynonanoic acid lactone	20.6	0.0000 *	0.318	0.7286
Benzenepropanol	35.0	0.0000 *	1.94	0.1489
Octanoic acid	15.4	0.0002 *	2.86	0.0624
Ethyl cinnamate	257	0.0000 *	1.20	0.3047
Cinnamyl acetate	42.1	0.0000 *	0.429	0.6521
Nonanoic acid	33.1	0.0000 *	2.07	0.1326
Thymol	41.2	0.0000 *	1.35	0.2638
Decanoic acid	42.6	0.0000 *	1.52	0.2239
2-nonenoic acid	31.0	0.0000 *	3.47	0.0354 *
Dihydromethyl jasmonate	106	0.0000 *	0.530	0.5904
Ɣ-Dodecalactone	72.4	0.0000 *	1.17	0.3139
Dodecanoic acid	56.3	0.0000 *	1.10	0.3348
Tetradecanoic acid	75.8	0.0000 *	0.436	0.6477

* Values are significant at *p* < 0.05.

**Table 3:** Mean relative areas and standard deviations of volatile identified by SBSE-GC-MS in Prickly pear Vinegar produced by surface culture method at 30 °C and 37 °C.

Compounds	30 °C	37 °C
	Mean ± SD	Mean ± SD
Ethyl acetate	0.1607 ± 0.1160	0.1733 ± 0.2798
1,3-dioxolane, 2,4,5-trimethyl-	0.0176 ± 0.0340 b	ND a
Diacetyl	0.0162 ± 0.0149 b	0.0022 ± 0.0042 a
Isobutyl acetate	0.0045 ± 0.0028	0.0050 ± 0.0147
Hexanal	0.0030 ± 0.0010 b	0.0023 ± 0.0011 a
2-methyl-1-propanol	0.0053 ± 0.0080 b	0.0000 ± 0.0001 a
Isoamyl acetate	0.0391 ± 0.0317	0.0412 ± 0.1034
2,6-dimethyl-4-heptanone	0.0058 ± 0.0055 b	0.0032 ± 0.0034 a
2-methyl-1-butanol	0.0411 ± 0.0568 b	0.0052 ± 0.0062 a
3-meth-1-butanol	0.0499 ± 0.0586 b	0.0054 ± 0.0066 a
Hexanoic acid, ethyl ester	0.0031 ± 0.0033	0.0039 ± 0.0049
Styrene	0.0003 ± 0.0003	0.0002 ± 0.0002
1-pentanol	0.0002 ± 0.0004 b	0.0000 ± 0.0001 a
Hexyl acetate	0.0022 ± 0.0036 b	0.0002 ± 0.0004 a
Acetoin	0.2520 ± 0.1984 b	0.0191 ± 0.0101 a
Acetol	0.0089 ± 0.0044	0.0081 ± 0.0087
Ethyl lactate	0.3537 ± 0.2461	0.3869 ± 0.0617
1-hexanol	0.0012 ± 0.0026 b	ND a
3-Hexen-1-ol, (E)-	0.0002 ± 0.0007 b	ND a
3-Hexen-1-ol, (Z)-	0.0004 ± 0.0007 b	ND a
Acetic acid	0.3995 ± 0.2952 b	0.0847 ± 0.0484 a
Octanoic acid, ethyl ester	0.0007 ± 0.0029	0.0007 ± 0.0034
*trans*-linalooloxide	0.0031 ± 0.0031 a	0.0127 ± 0.0013 b
*cis*-linalooloxide	0.0043 ± 0.0007 a	0.0068 ± 0.0009 b
1-Hexanol, 2-ethyl-	0.0025 ± 0.0010 a	0.0298 ± 0.0025 b
Cyclopentene	0.0002 ± 0.0003 b	ND a
Benzaldehyde	0.0088 ± 0.0073 a	0.0149 ± 0.0033 b
2,3-butanediol	0.0120 ± 0.0051 a	0.0184 ± 0.0071 b
Linalool	0.0022 ± 0.0018 b	0.0003 ± 0.0005 a
Isobutyric acid	0.0263 ± 0.0168 b	0.0063 ± 0.0020 a
1-octanol	0.0019 ± 0.0029 b	0.0001 ± 0.0005 a
Trans-2-cis-6-nonadienal	0.0003 ± 0.0005 b	ND a
Butanoic acid	0.0020 ± 0.0014 b	0.0001 ± 0.0003 a
Sulfide, allyl methyl	0.0049 ± 0.0034	0.0038 ± 0.0013
Isovaleric acid	0.0576 ± 0.0433 b	0.0101 ± 0.0048 a
1-nonanol	0.0025 ± 0.0051 a	0.0053 ± 0.0021 b
Butanedioic acid, diethyl ester	0.0204 ± 0.0112 a	0.0314 ± 0.0029 b
α-terpineol	0.0123 ± 0.0023 b	0.0042 ± 0.0007 a
2-Nonen-1-ol, (E)-	0.0027 ± 0.0060 b	0.0007 ± 0.0012 a
*cis*-6-nonenol	0.0297 ± 0.0395 b	0.0143 ± 0.0032 a
Benzyl acetate	0.0085 ± 0.0065 b	0.0029 ± 0.0003 a
β-Citronellol	0.0035 ± 0.0015 b	0.0021 ± 0.0007 a
Methyl salicylate	0.0019 ± 0.0012 b	0.0004 ± 0.0001 a
Ethyl phenylacetate	0.0218 ± 0.0245 b	0.0069 ± 0.0015 a
Phenethyl acetate	0.3165 ± 0.2540 b	0.0890 ± 0.0103 a
Hexanoic acid	0.0250 ± 0.0149 b	0.0114 ± 0.0018 a
Geraniol	0.0024 ± 0.0026 b	0.0002 ± 0.0003 a
*cis*-geranylacetone	0.0027 ± 0.0017	0.0026 ± 0.0051
Benzyl alcohol	0.0108 ± 0.0031	0.0112 ± 0.0009
Benzenepropanoic acid, ethyl ester	0.0094 ± 0.0064 b	0.0012 ± 0.0012 a
Phenylethyl alcohol	0.2801 ± 0.1939	0.2484 ± 0.0158
3-Phenyl-1-propanol, acetate	0.0134 ± 0.0207 b	0.0029 ± 0.0006 a
Phenol	0.0038 ± 0.0010 b	0.0031 ± 0.0005 a
4-hydroxynonanoic acid lactone	0.0489 ± 0.0078 a	0.0554 ± 0.0051 b
Benzenepropanol	0.0037 ± 0.0027 a	0.0062 ± 0.0006 b
Octanoic acid	0.1309 ± 0.0676 b	0.0899 ± 0.0099 a
Ethyl cinnamate	0.0047 ± 0.0013 b	0.0015 ± 0.0004 a
Cinnamyl acetate	0.0003 ± 0.0004 a	0.0006 ± 0.0001 b
Nonanoic acid	0.0572 ± 0.0321 b	0.0289 ± 0.0033 a
Thymol	0.0052 ± 0.0014 b	0.0037 ± 0.0006 a
Decanoic acid	0.0356 ± 0.0198 b	0.0153 ± 0.0046 a
2-nonenoic acid	0.0032 ± 0.0019 b	0.0015 ± 0.0005 a
Dihydromethyl jasmonate	0.0006 ± 0.0005 a	0.0020 ± 0.0008 b
Ɣ-dodecalactone	0.0206 ± 0.0053 b	0.0131 ± 0.0022 a
Dodecanoic acid	0.0137 ± 0.0085 b	0.0025 ± 0.0048 a
Tetradecanoic acid	0.0041 ± 0.0023 b	0.0007 ± 0.0011 a

Different letters in the same row indicate significant differences according to Tukey’s test (*p* < 0.05); ND: Not detected.

**Table 4:** Mean concentrations (ppm) and standard deviations of phenolic compounds identified by UPLC-DAD in different prickly pear matrices (juice, wine, vinegar). Results of analysis of variance taking into account the matrix.

Compounds	Juice	Wine	Vinegar	ANOVA	
	Mean ± SD	Mean ± SD	Mean ± SD	F Ratio	*p*-Value
Gallic acid	1.52 ± 0.042	1.24 ± 0.009	1.76 ± 0.471	1.48	0.2334
Hydroxy-tyrosol	4.34 ± 0.184	1.67 ± 0.147	2.85 ± 1.80	2.33	0.1035
Epigallocatechin	ND a	5.93 ± 0.172 b	8.26 ± 1.45 b	34.3	0.0000 *
Tyrosol	57.1 ± 2.54	42.4 ± 1.74	56.2 ± 9.62	2.10	0.1289
Vanillic acid	ND	ND	2.19 ± 0.776	0.492	0.6125
Syringic acid	1.66 ± 0.082 a	1.69 ± 0.035 ab	2.32 ± 0.376 b	5.65	0.0049 *
Ethyl gallate	3.57 ± 0.233 c	1.91 ± 0.164 b	ND a	17373	0.0000 *
m-Coumaric acid	0.564 ± 0.011 b	ND a	ND a	555,867	0.0000 *
Hesperidin	15.0 ± 3.63	4.39 ± 0.032	8.46 ± 2.06	13.8	0.0000 *
Naringenin	4.56 ± 0.879 c	2.47 ± 0.011 a	3.68 ± 0.908 b	2.73	0.0710
Protocatechualdehyde	0.956 ± 0.030 a	1.28 ± 0.027 ab	1.39 ± 0.165 b	7.42	0.0010 *
Caffeic acid	ND a	ND a	1.06 ± 0.100 b	220	0.0000 *
Ferulic acid	1.35 ± 0.065	1.33 ± 0.007	1.41 ± 0.250	0.154	0.8581
Quercetin	1.30 ± 0.441	1.13 ± 0.117	1.33 ± 0.309	0.452	0.6388
Cinnamic acid	ND	0.048 ± 0.020	0.125 ± 0.073	3.70	0.0286 *
p-Hydroxybenzoic acid	9.86 ± 0.469 b	1.33 ± 0.160 a	1.14 ± 0.450 a	370	0.0000 *

Different letters in the same row indicate significant differences according to Tukey’s test (*p* < 0.05). * Values are significant at *p* < 0.05; ND: Not detected.

**Table 5:** Analysis of variance considering the effect of temperature (30 °C/37 °C) and bacteria inoculum (*Acetobacter* (A)*/Gluconobacter* (G)/Mixture of bacteria (M)) on phenolic compounds of prickly pear vinegar produced by surface culture.

Compounds	Temperature (30 °C/37 °C)		Bacteria Inoculum (A/G/M)	
	F Ratio	*p*-Value	F Ratio	*p*-Value
Gallic acid	0.173	0.6836	5.95	0.0039 *
Hydroxy-tyrosol	115	0.0000 *	6.90	0.0017 *
Epigallocatechin	40.4	0.0000 *	2.90	0.0609
Tyrosol	19.2	0.0000 *	5.12	0.0080 *
Vanillic acid	1.74	0.1907	5.53	0.0056 *
Syringic acid	9.59	0.0027 *	4.46	0.0145 *
Hesperidin	7.60	0.0072 *	3.13	0.0491 *
Naringenin	107	0.0000 *	3.45	0.0363 *
Protocatechualdehyde	21.3	0.0000 *	4.00	0.0220 *
Caffeic acid	29.1	0.0000 *	0.583	0.5647
Ferulic acid	21.1	0.0000 *	1.24	0.2959
Quercetin	54.1	0.0000 *	1.08	0.3452
Cinnamic acid	21.2	0.0000 *	2.09	0.1299
p-Hydroxybenzoic acid	20.4	0.0000 *	1.59	0.2096

* Values are significant at *p* < 0.05.

**Table 6:** Mean concentrations (ppm) and standard deviations of phenolic compounds identified by UPLC-DAD in prickly pear vinegar produced by surface culture method at 30 °C and 37 °C, and for the three types of bacteria.

Compounds	30 °C	37 °C		*Acetobacter*	*Gluconobacter*	Mixture
	Mean ± SD	Mean ± SD		Mean ± SD	Mean ± SD	Mean ± SD
Gallic acid	1.76 ± 0.604	1.69 ± 0.156		1.78 ± 0.410 b	1.43 ± 0.527 a	2.10 ± 0.650 b
Hydroxy-tyrosol	2.71 ± 1.63 b	ND a		2.31 ± 1.36 a	4.05 ± 1.74 b	4.34 ± 2.94 b
Epigallocatechin	7.51 ± 1.49 a	8.97 ± 0.631 b		8.39 ± 1.27	7.16 ± 2.23	8.49 ± 2.34
Tyrosol	52.8 ± 12.1 a	60.3 ± 4.30 b		57.9 ± 9.26 b	47.8 ± 14.8 a	53.3 ± 7.37 ab
Vanillic acid	2.39 ± 0.738	1.38 ± 0.029		2.19 ± 0.776 b	ND a	ND ab
Syringic acid	2.24 ± 0.504 a	2.42 ± 0.168 b		2.38 ± 0.369 b	2.01 ± 0.601 a	2.14 ± 0.232 ab
Ethyl gallate	7.41 ± 1.77 a	9.74 ± 1.59 b		8.72 ± 2.08	7.50 ± 2.37	7.33 ± 2.11
m-Coumaric acid	2.82 ± 0.328 a	4.46 ± 0.484 b		3.63 ± 0.935 a	3.47 ± 1.16 ab	4.04 ± 0.676 b
Hesperidin	1.34 ± 0.198 a	1.44 ± 0.069 b		1.42 ± 0.150 b	1.22 ± 0.327 a	1.42 ± 0.256 ab
Naringenin	1.01 ± 0.082 a	1.11 ± 0.065 b		1.06 ± 0.083	0.992 ± 0.261	1.09 ± 0.168
Protocatechualdehyde	1.30 ± 0.233 a	1.53 ± 0.180 b		1.43 ± 0.238	1.26 ± 0.359	1.38 ± 0.373
Caffeic acid	1.13 ± 0.226 a	1.53 ± 0.204 b		1.35 ± 0.279	1.19 ± 0.412	1.38 ± 0.473
Ferulic acid	0.093 ± 0.045 a	0.159 ± 0.068 b		0.129 ± 0.067	0.100 ± 0.057	0.130 ± 0.131
Quercetin	1.36 ± 0.532 b	0.902 ± 0.144 a		1.15 ± 0.472	1.02 ± 0.376	1.34 ± 0.426
Cinnamic acid	1.76 ± 0.604	1.69 ± 0.156		1.78 ± 0.410 b	1.43 ± 0.527 a	2.10 ± 0.650 b
p-Hydroxybenzoic acid	2.71 ± 1.63 b	ND a		2.31 ± 1.36 a	4.05 ± 1.74 b	4.34 ± 2.94 b

For each variable (temperature and bacteria) different letters in the same row indicate significant differences according to Tukey’s test (*p* < 0.05); ND: Not detected.

## Data Availability

Data is contained within the article.
